# A satellite in the phage universe: isolation and characterization of six novel *Streptomyces* phages AndoPrime, Ando, Arkania, Jaresh, Tatooine, and Galidraan

**DOI:** 10.1093/femsmc/xtag048

**Published:** 2026-09-07

**Authors:** Moses Kabuu, Annika Ritzerfeld, Biel Badia Roigé, Samy Mechrouki, Maximilian K Nocke, Eva Davoudi, Tom Luthe, Julia Frunzke

**Affiliations:** Institute of Bio- and Geosciences, Biotechnology (IBG-1), Forschungszentrum Jülich, 52425 Jülich, Germany; Faculty of Mathematics and Natural Sciences, Institute of Microbial Interactions, Heinrich-Heine-Universität Düsseldorf, 40225 Düsseldorf, Germany; Department of Chemistry and Biotechnology, FH Aachen - University of Applied Sciences, 52428 Jülich, Germany; Institute of Bio- and Geosciences, Biotechnology (IBG-1), Forschungszentrum Jülich, 52425 Jülich, Germany; Faculty of Mathematics and Natural Sciences, Institute of Microbial Interactions, Heinrich-Heine-Universität Düsseldorf, 40225 Düsseldorf, Germany; Institute of Bio- and Geosciences, Biotechnology (IBG-1), Forschungszentrum Jülich, 52425 Jülich, Germany; Faculty of Mathematics and Natural Sciences, Institute of Microbial Interactions, Heinrich-Heine-Universität Düsseldorf, 40225 Düsseldorf, Germany; Ruhr University Bochum, Medical Faculty, Department of Translational and Computational Infection Research (TRACiR), 44801 Bochum, Germany; European Virus Bioinformatics Center (EVBC), 07743 Jena, Germany; Institute of Bio- and Geosciences, Biotechnology (IBG-1), Forschungszentrum Jülich, 52425 Jülich, Germany; Faculty of Mathematics and Natural Sciences, Institute of Microbial Interactions, Heinrich-Heine-Universität Düsseldorf, 40225 Düsseldorf, Germany; Institute of Bio- and Geosciences, Biotechnology (IBG-1), Forschungszentrum Jülich, 52425 Jülich, Germany; Ruhr University Bochum, Medical Faculty, Department of Translational and Computational Infection Research (TRACiR), 44801 Bochum, Germany; Institute of Bio- and Geosciences, Biotechnology (IBG-1), Forschungszentrum Jülich, 52425 Jülich, Germany; Faculty of Mathematics and Natural Sciences, Institute of Microbial Interactions, Heinrich-Heine-Universität Düsseldorf, 40225 Düsseldorf, Germany

**Keywords:** *Streptomyces*, actinobacteriophages, satellite phage

## Abstract

*Streptomyces* are soil-dwelling actinomycetes whose complex multicellular development is tightly linked to the production of bioactive specialized metabolites. The constant phage predation has continuously driven their evolution, whilst the complex development and metabolite secretion has co-evolved in response to the phage pressure shaping these interactions. Here, we report the isolation and characterization of six novel phages with one being a phage satellite, that were isolated as part of a public outreach program within the framework of the priority program SPP2330 involving local high schools. Five of the phages (i.e. AndoPrime, Ando, Arkania, Jaresh, and Tatooine) were isolated on *Streptomyces griseus*, while Galidraan was isolated on *Streptomyces galbus*. All phages exhibit a siphoviral morphotype except Arkania, which shows a podoviral morphotype. The isolates are virulent, with the exception of the satellite phage Ando, which is predicted to be a temperate phage. This distinct phage harbors a relatively small 13-kb genome encoding an integrase but lacks genes for lysis and tail proteins, rendering it dependent on its helper phage AndoPrime for productive infection. Collectively, this study expands the repertoire of characterized *Streptomyces* phages and provides new insights into their diversity and evolutionary dynamics within the complex host, shaping ecological interactions.

## Teaser

We report the isolation and characterization of six novel phages infecting *Streptomyces*, including a satellite-helper system. Additionally, this work highlights the strength of the participatory science of public outreach programs.

## Introduction

Bacteriophages (phages), the viruses of bacteria, are ubiquitously found in nature. With an estimated 10^31^ total particles and 10^24^ infections per second shaping bacterial communities in every environment, they are the most abundant biological entities on the planet (Clokie et al. 2011, Cobian Guemes et al. 2016, Mushegian 2020). Despite more than a century of phage research (Salmond and Fineran 2015), the morphological, genetic, and mechanistic diversity of bacteriophages and their host interactions were overlooked for a long time. Research efforts focused mainly on a fixed set of phages infecting *Escherichia coli* (mostly T phages) under standard conditions with additions of clinically relevant phages (Fruciano and Bourne 2007). Only recently, broadening the scope of phage research to new, more diverse hosts has led to the discovery of interesting phenomena such as the formation of a phage pseudonucleus (Chaikeeratisak et al. 2017), communication-based decision-making (Erez et al. 2017), and an unprecedented expansion of antiphage defense systems (Horvath and Barrangou 2010, Georjon and Bernheim 2023).

In addition to classical defense systems operating at the cellular level, multicellular bacterial strategies linked to complex lifestyles are attracting growing attention (Luthe et al. 2023); among the hosts of interest is *Streptomyces*, a genus of Gram-positive Actinomycetota that displays unique characteristics and plays an important role in diverse ecological niches (Barka et al. 2016). *Streptomyces* exhibits a complex multicellular lifestyle characterized by filamentous growth, the formation of a dense vegetative mycelium and the production of spores, formed on specialized reproductive structures called aerial hyphae (Flardh and Buttner 2009, Bush et al. 2015). One of the fundamental features of *Streptomyces* is its ability to produce a wide range of bioactive specialized metabolites (Alam et al. 2022). Transition through the different complex developmental stages is intricately coupled with the production of these specialized metabolites (Rigali et al. 2008, Yague et al. 2012, Tenconi et al. 2018), which include compounds used as pharmaceuticals, in agriculture and biotechnology (Demain 1999, Chater et al. 2010, Barka et al. 2016, Alam et al. 2022). Some of these specialized molecules were shown to confer antiphage properties, including DNA-intercalating anthracyclines (Kronheim et al. 2018) and aminoglycosides (Kever et al. 2022, 2024).

Phages infecting Actinomycetota, like *Streptomyces*, are called actinobacteriophages. To facilitate research and discovery on actinobacteriophage–host interactions, substantial efforts have been made in recent years to isolate, sequence, and characterize these phages (Hardy et al. 2020, Hatfull 2020, deCarvalho et al. 2023, Erdrich et al. 2024, Rackow et al. 2024). The Actinobacteriophage Database (PhagesDB) currently curates 6049 annotated actinobacteriophage genomes, 438 of which belong to those infecting *Streptomyces* (phagesdb.org, September 2026). Several public outreach programs have been established in recent years to promote public engagement alongside the expansion of phage arsenals. In addition to programs such as PHIRE (Phage Hunters Integrating Research and Education) or SEA-PHAGES (Science Education Alliance-Phage Hunters Advancing Genomics and Evolutionary Science) (Hatfull 2015), the priority program SPP 2330 (www.spp2330.de) established the lab course “Going Viral” addressing high school students to participate in science. In collaboration with the JuLab at Forschungszentrum Jülich, this outreach program presents six novel *Streptomyces* phages. AndoPrime, Ando, Arkania, Jaresh, Tatooine, and Galidraan expand the known diversity of actinobacteriophages by adding four new species across three existing subfamilies, while Ando and Galidraan each represent distinct species belonging to two novel genera. Notably, AndoPrime and Ando together constitute a unique satellite-helper phage system. Collectively, these phages infect at least seven different *Streptomyces* species.

## Materials and methods

### Bacterial strains and growth conditions

*Streptomyces* strains were inoculated from spore stocks and grown in liquid GYM medium (per liter: 4 g of glucose, 4 g of yeast extract, 10 g of malt extract, pH = 7.3) at 30°C and 170 rpm, unless mentioned otherwise. For growth on plates, GYM agar (adding 1.5% agar-agar and 0.8 g of CaCO_3_ per liter) was used. Infections on agar were performed with GYM soft-agar (0.4% agar-agar, w/o CaCO_3_).

### Phage isolation and propagation

Isolation of phages was done by using *Streptomyces griseus* DSM 40236 and *Streptomyces galbus* DSM 40089. Soil samples were dissolved in SM buffer (50 mM Tris-HCl, 100 mM NaCl, 8 mM MgSO_4_, pH = 7.5) and incubated for 3 h on a rock shaker at room temperature. The samples were centrifuged at 4000 g for 20 min and the supernatants were filtered through 0.2 µm pore size membrane filters (Sarstedt; Filtropur S, PES). Enrichment of phages was achieved by adding the filtered supernatant to 5x concentrated GYM medium (final concentration of 1x) and 500 µl of the respective *Streptomyces* strain. These enrichment cultures were incubated overnight at 30°C and 170 rpm before being centrifuged at 4000 g for 20 min. The supernatants were filtered again (0.2 µm pore size filters) and serial dilutions of the enriched supernatants were made in SM buffer. Different dilutions, including undiluted supernatant, were added to GYM soft-agar containing the respective host bacterium (OD_450_ = 0.4) and poured onto GYM agar plates. After incubation at 30°C, single plaques were picked and re-streaked three times with constant plaque morphology (Kauffman and Polz 2018) before considered as a phage isolate. These purified samples were harvested by adding 5 ml of SM buffer to the plate and incubating for 2 h on a rock shaker at room temperature. Liquid SM buffer and solubilized soft-agar were transferred into a falcon tube, centrifuged at 4000 g for 30 min, and the supernatants were filtered through 0.2 µm pore size filters to give a first lysate of the isolated phage.

### Plaque morphology development

Plaque morphology of the phages AndoPrime/Ando, Arkania, Jaresh, Tatooine, and Galidraan was assessed using the double-agar overlay method with a phage titer of 10^3^–10^4^ PFU/ml and host bacteria adjusted to OD_450_ = 0.4 in the top agar. Plates were incubated at 30°C for 48 h, while individual plaques were imaged after 24 and 48 h using a Nikon SMZ18 stereomicroscope equipped with NIS-Elements AR 5.3 software.

### Negative staining transmission electron microscopy of phage particles

For transmission electron microscopy (TEM) of individual phage particles, 3.5 µl of high titer (above 10^10^ PFU/ml) phage suspension was adsorbed onto a glow-discharged (15 mA, 30 s) carbon-formvar-coated copper grid (CF300-CU, 300 mesh) for 2 min. The grids were rinsed twice with distilled water, negatively stained for 30 s with 6 µl of 2% (w/v) uranyl acetate (UA), subsequently blotted at 45°C against a filter paper, and allowed to air-dry. The grids were imaged immediately or stored dry at room temperature, where they are stable for several weeks. Imaging of the negatively stained grids was performed on a Talos L120C G2 transmission electron microscope (Thermo Fisher Scientific, Dreieich, Germany) operated at 120 kV with a LaB6 electron source (Denka). The morphometric parameters of the phage particles, including capsid diameter and tail length, were measured using FIJI-ImageJ software (version 1.54 g).

### Host range determination

*Streptomyces* strains *S. griseus* (DSM 40236), *S. galbus* (DSM 40089), *S. bobili* (DSM 40481), *S. olivacues* (DSM 41536), *S. albus* (DSM 40313), *S. coelicolor* (DSM 112524), *S. xanthochromogenes* (DSM 40111), *S. cyaneofuscatus* (DSM 40148), *S. kasugaensis* (DSM 40189), and *S. venezuelae* (DSM 40230) were used in spot assays in order to determine the host range of the newly isolated phages. Serial dilutions of the phage lysates were made in SM buffer and 2 µl of each dilution were spotted on a respective bacterial lawn prepared as overlays from soft agar containing the host at OD_450_ = 0.4. Spotting was performed in duplicates. Efficiency of plating (EOP) was calculated relative to the isolation host.

### DNA isolation

To remove free DNA, 1 U/µl DNase I (Invitrogen, Carlsbad, CA, USA) was added to 2 ml of phage lysate. For phage DNA isolation, the Norgen Biotek Phage DNA Isolation Kit (Norgen Biotek, Thorold, Canada) was used according to the manufacturer’s instructions.

### DNA sequencing, genome assembly, gene prediction, and functional annotation

Quality control, library preparation, and sequencing of the isolated phage DNA were performed by GENEWIZ (Leipzig, Germany) using the Illumina MiSeq platform. Quality control of the reads was conducted and subsequent trimming for adapter sequences and low-quality reads (0.05) as well as ambiguous nucleotides was performed prior to *de novo* assembly using CLC genomics workbench 20.0.4 (Qiagen, Hilden, Germany). Resulting contigs were checked manually for length and coverage. PhageTerm (Garneau et al. 2017) was used for re-orientation and determination of phage termini class. The Pharokka pipeline (Bouras et al. 2023) was used for coding sequence (CDS) prediction and annotation of the phage genomes in an automated process with default parameters. Within Pharokka, CDSs were predicted with PHANOTATE (McNair et al. 2019), tRNAs were predicted with tRNAscan-SE 2.0 (Chan et al. 2021), tmRNAs were predicted with Aragorn (Laslett and Canback 2004), and CRISPRs were checked with CRT (Bland et al. 2007). Functional annotation was generated automatically by matching each CDS to the PHROGs (Terzian et al. 2021), VFDB (Chen et al. 2005), and CARD (Alcock et al. 2020) databases using MMseqs2 (Steinegger and Soding 2017) and PyHMMER (Larralde and Zeller 2023). Contigs were matched to their closest hit in the INPHARED (Cook et al. 2021) database using mash (Ondov et al. 2016). Afterwards, PhageAI (Tynecki et al. 2020) was used for prediction of the phage life style, which was confirmed by the presence or absence of integrase genes. Finished phage genomes were deposited in NCBI GenBank under the following accession numbers: PX421541 (Arkania), PX421542 (Tatooine), PX421543 (AndoPrime), PX421544 (Ando), PX421545 (Jaresh), and PX421546 (Galidraan).

### Genome comparison and classification

The newly isolated complete phage genome sequences were used for default basic local alignment and search tool (BLAST) searches against the NCBI nucleotide database [restricted to viruses (taxid: 10 239)] to find the closest related phages, which subsequently were used for genome comparison. Using VIRIDIC (Moraru et al. 2020) with default settings, pairwise comparison enabled calculation of the average nucleotide identities (ANIs) and classification according to standard taxon thresholds. Phylogenetic analysis of the phage terminase proteins was performed after alignment of sequences utilizing CLUSTALW (version 2.1) with a gap open penalty of 6 and a gap extension penalty of 4 (Thompson et al. 1994) (see Supplementary Fig. S1 for quality parameters computed with a custom Julia script, visualized in R) and the maximum likelihood tree was drawn in MEGA X (Kumar et al. 2018) using the LG algorithm (Le and Gascuel 2008) as best fit model with 500 bootstrap replicates. The tree was visualized with iTOL (Letunic and Bork 2021).

## Results and discussion

### Isolation and morphology

**Figure 1:**
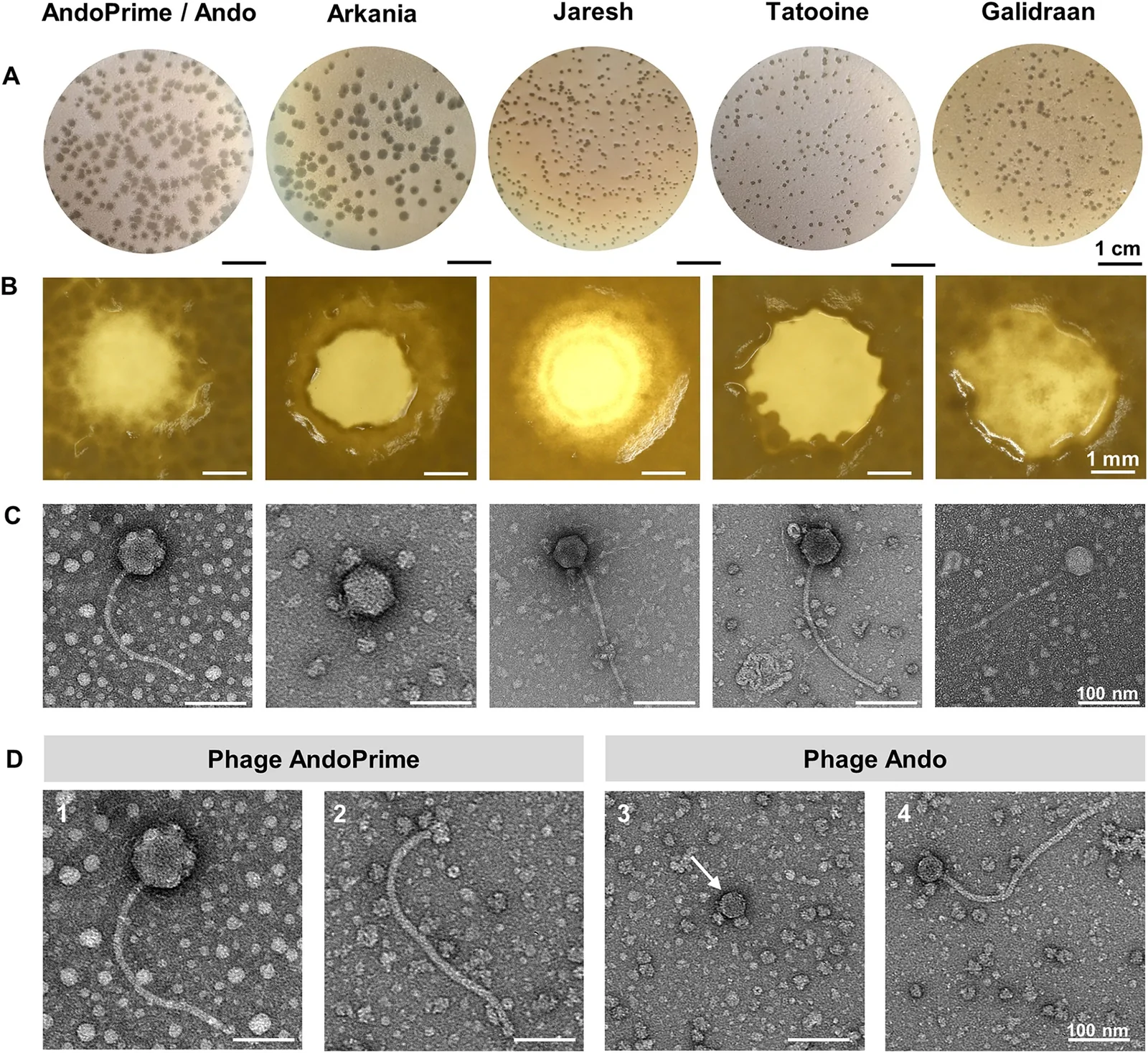
Morphology of novel *Streptomyces* phages. **(A)** Plaque morphologies of five phages on double-agar overlays. Phages AndoPrime/Ando, Arkania, Jaresh, and Tatooine isolated on *S. griseus* and phage Galidraan isolated on *S. galbus*. Images were taken after 48 h of incubation at 30°C. (scale bar: 1 cm). **(B)** Stereomicroscope images of representative plaques for each phage after 48 h (scale bar: 1 mm). **(C)** Transmission electron micrographs of negatively stained virions. **(D)** TEM images showing two distinct morphotypes. (**1**) A siphoviral particle with an icosahedral capsid (81 nm) and a long non-contractile tail (330 nm), (**2**) un-attached tail derived from the helper phage (330 nm), (**3**) smaller tailless capsid (47 nm) and (**4**) post-assembly attached small capsids to helper derived tails forming a siphoviral-like morphotype. Besides the distinct phage particles, small background particles are visible which likely represent residual lysate material, including host cell debris, membrane vesicles, proteins or salt precipitates. (uranyl acetate, scale bar: 100 nm).

Here we present the successful isolation of six new phages infecting *Streptomyces* species from environmental soil samples collected by students in the areas of Aachen and Düsseldorf, Germany. The phages AndoPrime, Ando, Arkania, Jaresh, and Tatooine were isolated on *Streptomyces griseus* and the phage Galidraan was isolated on *Streptomyces galbus*. All isolates formed clear plaques (Fig. 1a). Interestingly, both of the phages AndoPrime and Ando were isolated from the same sample in the same isolation experiment and could not be separated during infection experiments (Fig. 1a). The sizes of 10 randomly measured plaques per phage vary between the phages even on the same host. After 48 h of incubation, the phages AndoPrime/Ando and Arkania formed the largest plaques of 2.31 (±0.64) and 1.82 (±0.95) mm², whereas the phages Tatooine and Galidraan generated plaques of moderate sizes of 1.45 (±0.4) and 1.46 (±0.53) mm², respectively. Notably, the phage Jaresh formed the smallest plaques, averaging 0.73 (±0.13) mm² (Fig. 1b).

Analysis of the phages by TEM showed that the phages AndoPrime, Jaresh, Tatooine, and Galidraan possess long, non-contractile tails consistent with siphovirus morphology, whereas Arkania exhibits a short, non-contractile tail characteristic of podovirus morphology (Fig. 1c). These structural distinctions reflect functional differences in infection strategies: podoviruses are capable of rapid DNA delivery through their short tails following their adsorption (Casjens and Molineux 2012), while siphoviruses employ their elongated tails to recognize specific receptors on the host cell surface, facilitating adsorption and subsequent DNA injection (Wang et al. 2018). The phage Arkania, which exhibits the podoviral morphotype, had an average icosahedral capsid diameter of 59.2 nm and a short tail length of 15.7 nm. The other four phages showed a typical siphoviral morphotype with icosahedral capsid diameter ranging from 58 to 81 nm and non-contractile tails ranging between 242 and 332 nm (Table 1).

**Table 1:** Transmission electron microscopy (TEM). Measurements of phage capsid and tail dimensions: capsid diameters, and tail lengths. Single measurement events (n) reported in nanometers (nm) as mean (±) standard deviation.

Phage	Morphology	(n)	Capsid diameter (nm)	Tail length (nm)
Arkania	Podovirus	10	59.22 (±2.47)	15.74 (±2.33)
Jaresh	Siphovirus	11	77.81 (±2.45)	321.11 (±3.69)
Tatooine	Siphovirus	13	77.89 (±5.69)	332.11 (±8.92)
Galidraan	Siphovirus	15	58.55 (±1.99)	242.83 (±3.49)
Andoprime	Siphovirus	10	81.55 (±3.59)	330.03 (±3.03)
Ando	Siphovirus	12	47.38 (±5.98)	330.04 (±3.05)

Interestingly, the phages AndoPrime and Ando were inseparable, suggesting a potential helper-satellite interdependency. TEM analysis of the AndoPrime/Ando lysate showed a heterogenous population consisting of two distinct morphotypes: a typical siphoviral particle consistent with the helper phage AndoPrime (Fig. 1d, image 1), and unattached tails (Fig. 1d, image 2), alongside smaller tailless capsids (Fig. 1d, image 3). Quantitative measurements showed a significant difference between the two capsids with the smaller capsids measuring 47 nm while the phage AndoPrime capsids were about 81 nm (Table 1). This observation could be attributed to the smaller capsids of the satellite phage. In addition, there were numerous unattached tails derived from the helper phage whose sizes were consistent with the helper phage tails (Table 1). A notable observation was the presence of small capsids attached to helper-derived tails resulting in phage particles with a siphoviral-like morphotype (Fig. 1d, image 4). Importantly, the tail length of the siphoviral-like particles of Ando was consistent with that of the helper phage AndoPrime, despite differences in capsid size. This finding is particularly noteworthy because, as discussed in a subsequent section (Genomic features), the phage Ando does not encode genes for structural tail proteins. Together, the observation of the similarity in tail dimensions and the absence of tail structural proteins in the Ando genome supports our hypothesis that the satellite phage exploits the helper phage tails for DNA injection resulting in productive infection, likely through post-assembly attachment. Such systems have already been shown for other hosts like bacteria, which is expounded in subsequent sections.

### Host range of isolated phages

Despite phages being isolated on one particular host strain, infection of multiple strains or species is commonly observed among phages. These so-called host ranges were assessed by spotting our novel phages on bacterial lawns of 10 *Streptomyces* species available in the laboratory, including the two isolation hosts. The EOP was calculated for each phage-bacterium pair compared with the initial isolation host (Table 2). In contrast to previous work (Erdrich et al. 2024), all isolated phages are able to infect multiple *Streptomyces* species. AndoPrime and Ando yielded higher titers on *S. bobili* than on their isolation host *S. griseus* but performed worse on *S. galbus, S. olivaceus*, and *S. albus*. Similarly, Jaresh and Tatooine infected *S. bobili* better than *S. griseus*. Jaresh also yielded higher titers on *S. olivaceus*. Arkania has the smallest host range on the tested strains, showing low EOPs on *S. galbus* and *S. bobili* in addition to its isolation host. Moreover, Galidraan, isolated on *S. galbus*, is the only phage able to infect *S. xanthochromogenes*. No countable plaques could be observed for AndoPrime/Ando and Tatooine on *S. coelicolor* as well as Galidraan on *S. griseus*, but the bacterial lawn reacted with slight coloration in the spotted region. Finally, the strains *S. cyaneofuscatus, S. kasugaensis*, and *S. venezuelae* were not infected by any of the phages presented here.

**Table 2:** Host range determination. EOP was determined by spotting serial dilutions of phage lysates onto bacterial lawns and comparing plaque formation relative to the isolation host. An asterisk (*) indicates an absence of lysis but the presence of a morphological response of the bacterial lawn at the highest phage concentration.

	AndoPrime/Ando	Arkania	Jaresh	Tatooine	Galidraan
*S. griseus* DSM 40236	1.00*10^0^	1.00*10^0^	1.00*10^0^	1.00*10^0^	(*)
*S. galbus* DSM 40089	3.30*10^−2^	2.50*10^−5^	3.6*10^−3^	8.46*10^−1^	1.00*10^0^
*S. bobili* DSM 40481	5.67*10^0^	1.10*10^−4^	3.57*10^0^	1.62*10^1^	5.50*10^−2^
*S. olivacues* DSM 41536	1.40*10^−2^	–	1.43*10^0^	6.90*10^−4^	5.5*10^−8^
*S. albus* DSM 40313	4.00*10^−1^	–	2.29*10^−1^	1.67*10^1^	–
*S. coelicolor* DSM 112524	(*)	–	4.3*10^−5^	(*)	–
*S. xanthochromogenes* DSM 40111	–	–	–	–	4.5*10^−5^
*S. cyaneofuscatus* DSM 40148	–	–	–	–	–
*S. kasugaensis* DSM 40189	–	–	–	–	–
*S. venezuelae* DSM 40230	–	–	–	–	–

### Genomic features

Genome sizes and GC contents vary drastically between the six isolated phages (Table 3). Ando is the smallest phage with a genome size of 13 915 bp and a GC content of 51%. Arkania and Galidraan have medium sized genomes of around 45 and 43 kb, respectively, and the highest GC content of 60% each. On the upper range, genomes of Jaresh, AndoPrime, and Tatooine are the largest with over 100 kb and up to 131 kb as well as 132 kb, respectively. In contrast to our previous “Going Viral” study (Erdrich et al. 2024), the phages presented here are more diverse in terms of genome termination and packaging. For Jaresh, prediction using PhageTerm (Garneau et al. 2017) even resulted in a new and unknown mechanism. Except for Ando, all of the phages are predicted to have a virulent lifestyle and no integrases were found in their genomes.

**Table 3:** Genomic features. Basic genomic features of novel bacteriophages including genome size, GC content, number of open reading frames (ORF) predicted with Pharokka (Bouras et al. 2023), genome termini class determined using PhageTerm (Garneau et al. 2017), and lifestyle prediction by phage AI (Tynecki et al. 2020) and presence of integrase.

Phage name	Accession number	Isolation host	Genome size (bp)	GC content (%)	ORF number	Genome termini class	Lifestyle prediction
Arkania	PX421541	*S. griseus*	45 731	60	75	DTR (short)	Virulent
Tatooine	PX421542	*S. griseus*	132 518	47	270	DTR (short)	Virulent
AndoPrime	PX421543	*S. griseus*	131 713	49	271	DTR (long)	Virulent
Ando	PX421544	*S. griseus*	13 915	51	23	Headful (pac)	Temperate
Jaresh	PX421545	*S. griseus*	107 909	47	193	New	Virulent
Galidraan	PX421546	*S. galbus*	43 625	60	67	Cos (3’)	Virulent

Interestingly, the three phages with the largest genomes, AndoPrime, Jaresh, and Tatooine, harbor an extensive set of tRNA genes, comprising 36–45 tRNA-encoding genes that collectively cover the canonical 20 amino acids, except for AndoPrime and Tatooine, which both lack a tRNA for cysteine. tRNA genes for arginine, isoleucine, leucine, lysine, and threonine are overrepresented in these phages, each harboring three to five genes encoding tRNAs for those amino acids. Opposingly, Ando and Galidraan encode no tRNAs at all and Arkania has only 18 genes, each essential amino acid being represented once, except for cysteine and tyrosine, which are missing completely. Additionally, the phages presented here harbor many genes related to DNA and RNA metabolism, repair, and replication. For example, DNA primase, DNA polymerase (replicative) helicases, multiple DNA binding proteins, holliday junction resolvases and endo- as well as exonucleases are present in all phages, except for Ando. Further noticeable genes include those coding for the WhiB-like transcriptional regulator (Jaresh, Tatooine, AndoPrime), FtsK/SpoIIIE-like protein (Jaresh, AndoPrime), and Lsr2-like nucleoid-associated protein (Tatooine, AndoPrime, Ando). WhiB-type regulators were previously reported as the most abundant regulatory proteins encoded by actinobacteriophages (Hardy et al. 2020, Sharma et al. 2021, Chen and Banfield 2024, Erdrich et al. 2024).

Especially in *Streptomyces*, where WhiB-like regulators and FtsK-like proteins are involved in multicellular development, phage infection was shown to be affected by the developmental state of the host (Bush 2018, Luthe et al. 2023). Lsr2-like proteins are widespread xenogeneic silencing proteins in actinobacteria and frequently encoded by their phages as well (Hardy et al. 2020, Sharma et al. 2021, Chen and Banfield 2024, Erdrich et al. 2024). Several recent studies reveal significant functional diversification, including important roles in phage host interaction (Pfeifer et al. 2019), ranging from the support of phage propagation (Dulberger et al. 2023), silencing of cryptic prophages (Pfeifer et al. 2016), to defense against AT-rich phages (Badia Roigé et al. 2026).

In Jaresh, Tatooine, and AndoPrime, RIIA and RIIB lysis inhibitor proteins are encoded, which seem to play a role for membrane integrity during phage replication (Wong et al. 2021). A distinguishing feature of phage Galidraan is that it encodes an RNA-dependent RNA polymerase typically only relevant to the replication and transcription of RNA viruses (Zanotto et al. 1996, Choi 2012, Alphonse and Ghose 2017). Next to many more different genes, most of the predicted ORFs are of unknown function, resulting in overall 60% (Ando) to 84% (Arkania) of genes being annotated as coding for hypothetical proteins. This is comparable to dsDNA phages in general, where more than 50% of genes are annotated as hypothetical, while rising to 70% for actinobacteriophages (Mahmoudabadi and Phillips 2018, Hatfull 2020).

### Average nucleotide identity, phylogenetic analysis, and taxonomy

**Figure 2:**
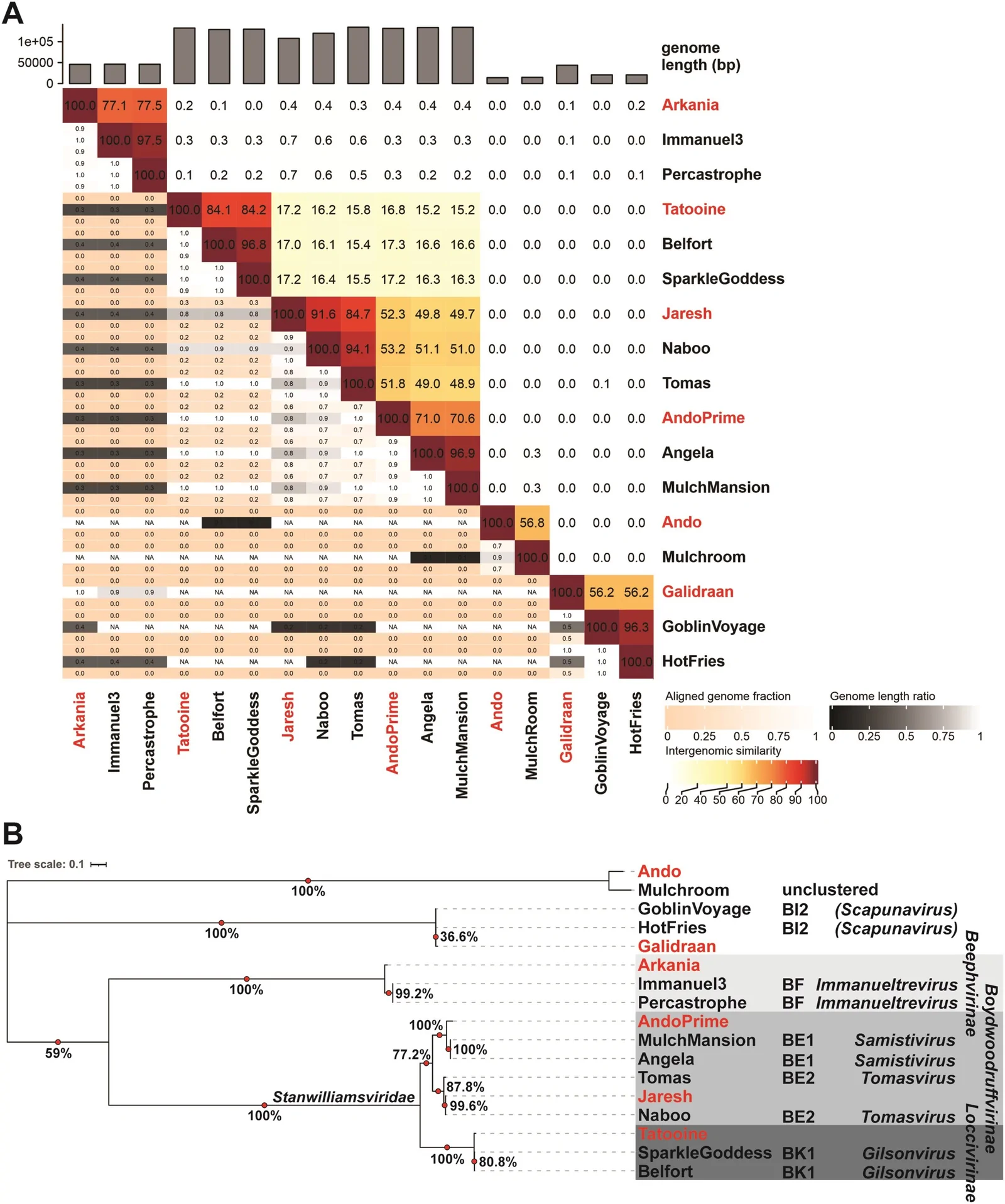
Average nucleotide identity and phylogenetic analysis. Novel phages are labeled in red; sequences were obtained from NCBI based on relatedness to our isolated phages. **(A)** Average nucleotide identity (ANI) analysis was performed using VIRIDIC with default settings. The heatmap shows intergenomic similarity values (right half) and alignment indicators (left half) as well as sequence length (top). The alignment indicators show aligned fraction of genome 1, genome length ratio, and aligned fraction of genome 2 (top to bottom). Colour codes are according to presented legends. **(B)** Phylogenetic analysis of phage-encoded terminase proteins. Amino acid sequences were aligned using CLUSTALW and the best fit model LG was used with 500 bootstrap replicates generating a maximum-likelihood tree (for details see Methods). Actinobacteriophage clusters from phagesdb.org are assigned. Red dots indicate bootstrap support. The tree scale represents 0.1 substitutions per site and the tree was visualized with iTol.

Searches using BLAST revealed a set of 11 closest relatives to our novel isolates that were obtained from NCBI and used for further ANI and phylogenetic analyses. According to this, all phages are new species and belong to the class of *Caudoviricetes* (Fig. 2a). Arkania belongs to the genus *Immanueltrevirus* within the *Beephvirinae* subfamily as it is closest related to phage Percastrophe (77.5%, MG663583.1). Tatooine is closest related to Sparklegoddess (84.2%, MH590589.1) and therefore belongs to the family of *Stanwilliamsviridae*, subfamily *Loccivirinae*, and genus *Gilsonvirus*. Phage Naboo, previously isolated in our “Going Viral” course (Erdrich et al. 2024), is the closest relative to Jaresh (91.6%, PP171442.1). Both belong to the *Stanwilliamsviridae*, subfamily *Boydwoodruffvirinae*, and the genus *Tomasvirus*. AndoPrime just barely fits within an existing genus. According to its closest relatives Angela (71.0%, ON970591.1) and MulchMansion (70.6%, MT897905.1), AndoPrime belongs to the genus *Samistivirus* in the *Stanwilliamsviridae* family and *Boydwoodruffvirinae* subfamily. Phylogenetic analysis of the terminase proteins of all 17 phages confirms ANI results and the maximum-likelihood tree supports the aforementioned taxonomy (Fig. 2b).

The *Stanwilliamsviridae* family is a large group of *Streptomyces*-infecting phages already expanded with all five phages from our last “Going Viral” project (Erdrich et al. 2024). The phages in this family harbor many genes relevant for DNA metabolism and replication. Those genes include DNA primases, DNA polymerases, DNA binding proteins and helicases. All these genes can be found in the here-isolated phages Tatooine, Jaresh, and AndoPrime falling into this family and are thought to be essential for the formation of the replisome, described already for T4 and T7 phages (Benkovic and Spiering 2017, Magill et al. 2018).

Based on the criteria of the ICTV’s subcommittee (Turner et al. 2021), we suggest the phages Ando and Galidraan will form new genera as their closest found relatives share an average nucleotide identity lower than 70% (56.8% Ando with Mulchroom, OQ784832.1; 56.2% Galidraan with HotFries, MH155869.1) and terminase protein phylogeny shows well-supported monophyletic clustering. Furthermore, when additional closely related phages are discovered, Galidraan and Ando could potentially form new subfamilies with the known genus of *Scapunavirus* and unassigned Mulchroom, respectively.

### A new satellite-helper phage system infecting *Streptomyces*

The most distinct phage of the new isolates presented here is Ando. It is particularly smaller and predicted to be temperate due to encoding an integrase. Interestingly, Ando could only be isolated together with AndoPrime and does not harbor lysis genes. In addition to the integrase and 14 hypothetical proteins, the genome encodes a terminase, two transcriptional regulators, and the DNA-bridging protein Lsr2. Among the structural proteins, only capsid proteins are present, while proteins required for tail formation are entirely absent. TEM analysis of the AndoPrime/Ando lysate showed a heterogenous population consisting of two distinct morphotypes, where the smaller tailless capsids attributed to the satellite phage attached to the helper- derived tails result in phage particles with a siphoviral-like morphotype, as shown in Fig. 1d, image 4. These observations support the hypothesis that Ando and AndoPrime form a satellite-helper system, in which the satellite phage Ando may depend on helper-derived tails for host cell attachment and DNA injection; however, this model remains to be confirmed experimentally.

Such systems are already known in other host bacteria like *E. coli*. Here, one of the best described satellite-helper systems is the P2-P4 system (Lindqvist et al. 1993, Delesalle et al. 2016). This system consists of the helper phage P2 and plasmid DNA P4. Another example of satellites are the so-called phage-induced chromosomal islands (PICIs). Although they fall under the definition of a virus (non-organismal, ultrafilterable), these mobile genetic elements are classified as satellite DNA (Koonin et al. 2021). Similar to the PICIs are the capsid forming phage-induced chromosomal islands, a family of satellite phages present across multiple bacterial species and genera (He et al. 2025). These entities assemble their own capsids and package their DNA independently. Their capsids are however tailless and non-infective, relying entirely on phage tails for transfer (He et al. 2025). This mirrors our observation, where the satellite phage Ando similarly forms tailless capsids that subsequently attach to the helper phage tails for productive infection.

Satellite viruses on the other hand are defined as mobile genetic elements encoding one or more proteins required for virion formation, but are dependent on a helper virus for genome replication (Koonin et al. 2021). Despite some satellites in bacterial hosts being referred to as satellite phages, they do not fall under this criterion. However, Ando fits this definition as it encodes a portal, a capsid, and a scaffolding protein, but lacks lysis and replication genes. True satellite phages were only recently discovered: *Acinetobacter* satellite phage Aci01-2-Phanie and helper phage Aci01-1, as well as *Streptomyces* helper-satellite systems MindFlayer/MiniFlayer and MulchMansion/Mulchroom (deCarvalho et al. 2023, Pourcel et al. 2024). Interestingly, MulchMansion and Mulchroom are the closest relatives to our isolated AndoPrime and Ando, further underlining the characterization as a satellite-helper phage system. Both Ando and Mulchroom are temperate and encode their own capsid structure but are dependent on the tail of their helper (deCarvalho et al. 2023). This fits the strategy of the satellite phage residing as an integrated prophage with its host until the virulent helper phage infects and provides replication, virion, and lysis components for the satellite to propagate and spread.

## Conclusion

The six newly isolated *Streptomyces* phages presented here emphasize both the extraordinary diversity of bacteriophages and the large fraction of unexplored viral dark matter that remains in nature (Dion et al. 2020). Their characterization revealed high levels of functional novelty, several new taxonomic assignments at the species and genus levels, and a previously unrecognized satellite-helper phage system that broadens our view of phage strategies in complex bacterial hosts. Together, these findings reinforce the importance of *Streptomyces* phage research for uncovering new principles of virus–host interaction and highlight the exceptional scientific value of outreach programs as engines for both education and discovery.

## Supplementary Material

xtag048_Supplemental_Files

## Data Availability

Phage genomes are deposited in NCBI GenBank under the following accession numbers: PX421541 (Arkania), PX421542 (Tatooine), PX421543 (AndoPrime), PX421544 (Ando), PX421545 (Jaresh), and PX421546 (Galidraan).
