# Supplementary material for: A satellite in the phage universe: isolation and characterization of six novel Streptomyces phages AndoPrime, Ando, Arkania, Jaresh, Tatooine, and Galidraan
Source: FEMS Microbes. 2026 Sep 7;7:xtag048. doi: 10.1093/femsmc/xtag048 (PMC13587820; doi:10.1093/femsmc/xtag048)
Supplement: Supplementary file 1 [file xtag048_supplemental_files.zip › FEMSMC-2026-048.R2 supplementary data - reviews and author response.docx]

**Reviews of manuscript FEMSMC-2026-048**

**Reviewer 1: Stephanie Bury-Moné**

**Recommendation: Accept**

This study presents the isolation and characterization of six novel Streptomyces phages, including a satellite phage (Ando), as part of the public outreach program "Going Viral", which engages high school students in scientific discovery. Five phages were isolated using S. griseus (AndoPrime, Ando, Arkania, Jaresh, and Tatooine), while Galidraan was isolated using S. galbus. The phages exhibit diverse taxonomic assignments at the species and genus levels, and their host tropism was evaluated. Morphologically, most are siphoviruses, except for Arkania, which displays a podoviral morphotype.

A highlight of this work is the characterization of Ando, a temperate satellite phage with a compact 13 kb genome. Notably, Ando lacks lysis genes and tail proteins, depending on its helper phage, AndoPrime, for productive infection. Together, Ando and AndoPrime represent a unique satellite-helper phage system, offering valuable insights into phage-host interactions.

This study expands the known diversity of Streptomyces phages and contributes to unraveling the "viral dark matter" in nature. The manuscript is well-written, and the experiments adhere to the gold standard. I have no major concerns.

I particularly appreciate the emphasis on the scientific value of outreach programs, which serve as powerful tools for both education and discovery.

Minor suggestions:

- In the microscopy images, consider adding a brief explanation in the figure legend to clarify the small entities visible in the background.

- In Table 2, I recommend formatting numerical values in scientific notation (e.g., 0.000025 → 2.5 × 10⁻⁵) for improved readability.

**Reviewer 2: Vera van Noort, KU Leuven**

**Recommendation: Reject**

This work finds 6 novel Streptomyces phages of which one is a satellite-helper phage.

A lot of methodological details are missing. The conclusion that the novel phages are monophyletic is not supported by the results.

Methods: please explain in more detail:

Functional annotation was generated by matching each CDS to the PHROGs [36], VFDB [37] and CARD [38] databases using MMseqs2 [39] and PyHMMER [40].

How was an annotation derived? Did you copy the annotation for the top-hit, how was it consolidated between different sources?

For many phage genomes, the functional annotation is a bottleneck. What is the fraction of genes for which a function could be annotated? How does this compare to other phages in phageDB?

The BLAST analysis is unclear. What was used as input for the BLAST searches? The complete genomes? Which parameters, thresholds were used.

For the ANI analysis, the tool is called VIRIDIC (not VIRIDICT). In the legend of Figure 2 a description is missing on what is represented in each part of Figure panel A. Also the settings for VIRIDIC analysis are missing.

A quick look at the database entries at NCBI results in a seqconfig error. Please check the NCBI submissions for correctness.

For the terminase phylogeny, since the ANI is so low, please provide the length of the Multiple sequence alignment and some quality parameters of the alignment (percentage without gaps). The tree needs more quality control. **Bootstrap support above 50% is not enough to conclude that these are monophyletic, this should be at least 90%.**

In Figure 2B it looks like the authors have covered some information with a white rectangle with grey outline. Please remove this.

- Please remove any mentioning of the public outreach program in the methodology and abstract and results. Restrict this to the acknowledgement/funding section.

- Introduction: Please remove Several public outreach programs

have been established in recent years to promote public engagement alongside the expansion of phage

arsenals. In addition to programs such as PHIRE (Phage Hunters Integrating Research and Education)

or SEA-PHAGES (Science Education Alliance-Phage Hunters Advancing Genomics and Evolutionary Science) [28], the priority program SPP 2330 (www.spp2330.de) established the lab course “Going Viral” addressing high school students to participate in science. In collaboration with the JuLab at

Forschungszentrum Jülich, this outreach program presents

Abstract: Remove "that were isolated as part of a public outreach program within the

framework of the priority program SPP2330 involving local high schools"

Methods: Remove "by the students near their school, home or the Forschungszentrum Jülich "

Results: please remove "The outreach program “Going Viral” is dedicated to give high school students the opportunity to

participate in scientific work and discovery. In the frame of the DFG-funded priority program SPP 2330 (“New concepts in prokaryotic virus-host interaction”, www.spp2330.de), together with the JuLab of

Forschungszentrum Jülich, the students "

**Associate Editor Recommendation: Major Revision**

Thank you for submitting this manuscript to FEMS Microbes. The reviewers recognized several strengths of the study, including the isolation and characterization of novel Streptomyces phages and the potentially interesting Ando/AndoPrime satellite-helper phage system. I agree with the reviewers that the manuscript is within the scope of the journal and could make a useful contribution to the field.

However, the manuscript is not yet suitable for acceptance in its current form. The major issues are primarily related to reproducibility of the genomic analyses, clarity of the taxonomic and phylogenetic interpretations, verification of sequence-data deposition, and calibration of some of the mechanistic claims. In particular, the Methods should provide sufficient detail for the annotation, BLAST, VIRIDIC, ANI, and phylogenetic analyses to be reproduced, including relevant inputs, parameters, thresholds, and criteria for interpreting results. The authors should also verify that all GenBank records are complete and accessible.

The Ando/AndoPrime system is a strength of the manuscript, but some statements currently go beyond what the presented data directly demonstrate. The authors should either provide additional support for strong mechanistic claims or revise the language throughout to make clear where the data are suggestive or consistent with a satellite-helper relationship rather than definitive proof of the proposed mechanism.

The authors should also revise the framing of the outreach program. It is appropriate to acknowledge the provenance of the isolates and the contribution of participating students where relevant, but broad claims about the educational or outreach impact of the program should be removed or substantially limited unless directly evaluated in the study.

Finally, please address the reviewers’ specific comments regarding figure clarity, table formatting, VIRIDIC nomenclature, phylogenetic support, and presentation of the microscopy and comparative genomics data. A revised manuscript should be accompanied by a detailed point-by-point response explaining how each reviewer comment has been addressed.

I am therefore inviting a major revision. A substantially revised manuscript that addresses these concerns will be considered further, but acceptance cannot be guaranteed at this stage.

**EIC Decision: Major Revision**

**Author's Response to Decision Letter for (FEMSMC-2026-048)**

Dear Prof. Dr. Scott,

Thank you for the opportunity to revise our manuscript. We have carefully addressed the reviewers’ comments and have substantially revised the manuscript. We provide a detailed point-by-point response to all reviewer comments.

In particular, we expanded the methodological descriptions to improve reproducibility of the genomic annotation, BLAST, VIRIDIC, ANI, and phylogenetic analyses; verified the accessibility and completeness of all GenBank records; revised the taxonomic and mechanistic interpretations; clarified the presentation of the Ando/AndoPrime satellite-helper system; and improved the figures, legends, and table formatting.

Please note, we do not only provide the data, but also will deposit all virus isolates at the DMSZ to make the biological material available to the community.

We also reduced and refocused the discussion of the outreach program. However, we would like to mention the programme in the manuscript and don’t think that this has a negative impact on the presented science.

We’d like to point out, that Dr. Nocke was added as an additional co-author as his expertise in bioinformatics was of invaluable help for the revision of the manuscript. To address the reviewers comments best, he worked on refining our initial multiple sequence alignment of the phage terminase proteins, generated the new supplementary figure S1 and helped in quality checking our computational analyses in general. With that he provided the basis for updating to an improved Figure 2, to which he contributed, too. Additionally, he helped in reviewing and editing the manuscript. These substantial inputs are also recognized in the Author Contributions stated at the end of the manuscript.

We appreciate the constructive feedback, which has helped us improve the clarity and rigor of the manuscript. We hope that the revised version is now suitable for further consideration in FEMS Microbes.

Thank you for your consideration.

Sincerely,

Tom Luthe and Julia Frunzke

**Reviewer 1:**

Comments to the Author

This study presents the isolation and characterization of six novel *Streptomyces* phages, including a satellite phage (Ando), as part of the public outreach program "Going Viral", which engages high school students in scientific discovery. Five phages were isolated using *S. griseus* (AndoPrime, Ando, Arkania, Jaresh, and Tatooine), while Galidraan was isolated using *S. galbus*. The phages exhibit diverse taxonomic assignments at the species and genus levels, and their host tropism was evaluated. Morphologically, most are siphoviruses, except for Arkania, which displays a podoviral morphotype.

A highlight of this work is the characterization of Ando, a temperate satellite phage with a compact 13 kb genome. Notably, Ando lacks lysis genes and tail proteins, depending on its helper phage, AndoPrime, for productive infection. Together, Ando and AndoPrime represent a unique satellite-helper phage system, offering valuable insights into phage-host interactions.

This study expands the known diversity of *Streptomyces* phages and contributes to unraveling the "viral dark matter" in nature. The manuscript is well-written, and the experiments adhere to the gold standard. I have no major concerns.

I particularly appreciate the emphasis on the scientific value of outreach programs, which serve as powerful tools for both education and discovery.

In the microscopy images, consider adding a brief explanation in the figure legend to clarify the small entities visible in the background.

**Answer:** A short description has been added to the Figure 1 legend:

“Besides the distinct phage particles, small background particles are visible which likely represent residual lysate material, including host cell debris, membrane vesicles, proteins or salt precipitates”

In Table 2, I recommend formatting numerical values in scientific notation (e.g., 0.000025 → 2.5 × 10⁻⁵) for improved readability.

**Answer:** We changed the values in table 2 to scientific notation.

**Reviewer 2:**

Comments to the Author

This work finds 6 novel *Streptomyces* phages of which one is a satellite-helper phage.

A lot of methodological details are missing. The conclusion that the novel phages are monophyletic is not supported by the results.

Methods: please explain in more detail: Functional annotation was generated by matching each CDS to the PHROGs [36], VFDB [37] and CARD [38] databases using MMseqs2 [39] and PyHMMER [40]. How was an annotation derived? Did you copy the annotation for the top-hit, how was it consolidated between different sources?

**Answer:** The CDS prediction and annotation process is automated within the Pharokka tool as outlined in the respective publication ([31]: https://academic.oup.com/bioinformatics/article/39/1/btac776/6858464) and was used by us with default parameters. Our methods section summarizes the basic steps of the pipeline briefly. Main source for annotation is the PHROGs database with additional, specialized databases on virulence factors (VFDB) and antimicrobial resistance genes (CARD) supporting the process. Results are manually checked for plausibility using BLASTp. We reworked some details in this section of the text to improve understanding and clarity:

“PhageTerm [30] was used for re-orientation and determination of phage termini class. The Pharokka pipeline [31] was used for coding sequence (CDS) prediction and annotation of the phage genomes in an automated process with default parameters. Within Pharokka, CDS were predicted with PHANOTATE [32], tRNAs were predicted with tRNAscan-SE 2.0 [33], tmRNAs were predicted with Aragorn [34] and CRISPRs were checked with CRT [35]. Functional annotation was generated automatically by matching each CDS to the PHROGs [36], VFDB [37] and CARD [38] databases using MMseqs2 [39] and PyHMMER [40]. Contigs were matched to their closest hit in the INPHARED [41] database using mash [42]. Afterwards, PhageAI [43] was used for prediction of the phage life style which was confirmed by the presence or absence of integrase genes.”

For many phage genomes, the functional annotation is a bottleneck. What is the fraction of genes for which a function could be annotated? How does this compare to other phages in phageDB?

**Answer:** That is true. Pharokka is inspired by Prokka but specifically tailored to bacteriophages to improve CDS prediction and annotation. For our here described phages, between 60% and 84% of CDS could not be functionally annotated, resulting in hypothetical proteins (see section “Genomic Features” in the Results and Discussion). In general, more than 50% of all dsDNA bacteriophage genes are annotated as hypothetical (<https://elifesciences.org/articles/31955>). For actinobacteriophages, this percentage rises to 70% (<https://www.annualreviews.org/content/journals/10.1146/annurev-virology-122019-070009>). We included this information in the manuscript.

The BLAST analysis is unclear. What was used as input for the BLAST searches? The complete genomes? Which parameters, thresholds were used.

**Answer:** Thanks for pointing that out. We added more detail to the section “Genome comparison and classification” in the methods:

“The newly isolated complete phage genome sequences were used for default BLAST (basic local alignment and search tool) searches against the NCBI nucleotide database (restricted to viruses (taxid: 10239)) to find the closest related phages which subsequently were used for genome comparison.”

For the ANI analysis, the tool is called VIRIDIC (not VIRIDICT). In the legend of Figure 2 a description is missing on what is represented in each part of Figure panel A. Also the settings for VIRIDIC analysis are missing.

**Answer:** Thanks for spotting this typo, we corrected the spelling. We improved the legend of Figure 2A as follows:

“**(A)** Average nucleotide identity (ANI) analysis was performed using VIRIDIC with default settings. The heatmap shows intergenomic similarity values (right half) and alignment indicators (left half) as well as sequence length (top). The alignment indicators show aligned fraction of genome 1, genome length ratio, and aligned fraction of genome 2 (top to bottom). Colour codes are according to presented legends.

A quick look at the database entries at NCBI results in a seqconfig error. Please check the NCBI submissions for correctness.

**Answer:** All entries were checked for accessibility with both the accession number and the name of the phages. Entries in GenBank for all six phages are complete, accessible, downloadable and could be opened with a text editor and, for example, Snapgene without any issues. We could not find the mentioned error.

For the terminase phylogeny, since the ANI is so low, please provide the length of the Multiple sequence alignment and some quality parameters of the alignment (percentage without gaps). The tree needs more quality control. **Bootstrap support above 50% is not enough to conclude that these are monophyletic, this should be at least 90%.**

**Answer:** Thanks a lot for pointing this out. We were looking closer into our multiple sequence alignment and were able to refine it. Quality parameters can be seen in the new supplementary material figure S1. You can see the coverage of sequences for each position of the MSA in A, where blue indicates number of sequences with amino acid information at the respective position of the alignment and yellow indicates sequences with gaps. In B, the Shannon entropy indirectly indicates information content (hence diversity) on the left axis (shades of red) with a lower value indicating lower diversity. On the right axis conservation of the most frequent amino acid at the relevant position is indicated in green.

With this refined, high-quality MSA, we have reworked the maximum-likelihood tree in Figure 2B (best fit model LG). Red dots are now accompanied by the exact respective bootstrap values. This shows strong bootstrap support (>90%) for all of our newly isolated phages and confirms our previous conclusions regarding new species and genera nicely. Additionally, this reflects the known taxonomy better than the previous tree.

Both the Methods section referring to constructing the alignment and maximum-likelihood tree as well as the legend to Figure 2B were reworked to represent these changes.

In Figure 2B it looks like the authors have covered some information with a white rectangle with grey outline. Please remove this.

**Answer:** As we reworked Figure 2 due to the new phylogenetic analysis, we think that this impression is resolved now. We are sorry for the confusion.

Please remove any mentioning of the public outreach program in the methodology and abstract and results. Restrict this to the acknowledgement/funding section.

**Answer:** As we as the scientific community thrive through education, active participation and communication with the public and next generation of young scientists, we advocate for a greater visibility of such outreach programs. Our outreach program was essential to isolating and characterizing the here presented phages. As also mentioned by reviewer 1, this is highly appreciated and needs further visibility. Nonetheless, we agree that public outreach is not the focus of the scientific work in this research article. Therefore, we will remove the parts referring to it from the methodology and results sections but will still shortly mention the program in the introduction.

Introduction: Please remove Several public outreach programs have been established in recent years to promote public engagement alongside the expansion of phage arsenals. In addition to programs such as PHIRE (Phage Hunters Integrating Research and Education) or SEA-PHAGES (Science Education Alliance-Phage Hunters Advancing Genomics and Evolutionary Science) [28], the priority program SPP 2330 ([www.spp2330.de](http://www.spp2330.de)) established the lab course “Going Viral” addressing high school students to participate in science. In collaboration with the JuLab at Forschungszentrum Jülich, this outreach program presents

**Answer:** We respectfully prefer to retain this paragraph in the Introduction. As outlined in our previous response, we believe that mentioning outreach initiatives such as PHIRE, SEA-PHAGES, and the “Going Viral” course is relevant in the context of expanding phage research and education. These programs help increase the visibility of phage research, promote public engagement, and highlight the importance of outreach activities for the broader scientific community. We therefore do not see a scientific reason to remove this section.

Abstract: Remove "that were isolated as part of a public outreach program within the
 framework of the priority program SPP2330 involving local high schools"
 Methods: Remove "by the students near their school, home or the Forschungszentrum Jülich "

**Answer:** We will keep the part in the abstract as already explained, to increase visibility and underline the importance of such outreach programs for the scientific community. In the methods section, “Phage isolation and propagation”, reference to the students was deleted:

“Soil samples were dissolved in SM buffer (50 mM Tris-HCl, 100 mM NaCl, 8 mM MgSO_4_, pH = 7.5) and incubated for 3 h on a rock shaker at room temperature.”

Results: please remove "The outreach program “Going Viral” is dedicated to give high school students the opportunity to participate in scientific work and discovery. In the frame of the DFG-funded priority program SPP 2330 (“New concepts in prokaryotic virus host interaction”, [www.spp2330.de](http://www.spp2330.de)), together with the JuLab of Forschungszentrum Jülich, the students "

**Answer:** We agree on removing this part from the results section. The section now starts as follows:

“Here we present the successful isolation of six new phages infecting *Streptomyces* species from environmental soil samples collected by students in the area of Aachen and Düsseldorf, Germany.”

**Reviews of manuscript FEMSMC-2026-048.R1**

**Reviewer 1: Stephanie Bury-Moné**

**Recommendation: Accept**

The authors have addressed the points raised during the review process very well. In my opinion, the manuscript is now suitable for publication.

**Reviewer 2: Vera van Noort, KU Leuven**

**Recommendation: Accept**

The authors have made a good effort to clarify the parts that were unclear and redo the phylogenetic analysis so that the conclusions are now supported by the results. I agree it can now be accepted for publication

**Reviewer 3: Eleanor Jameson, Bangor Univ**

**Recommendation: Minor Revision**

This paper reports the isolation and characterisation of six Streptomycees bacteriophages. The paper is well written and provides clear methods. It details the host range, morphology and outlines the genomic content of these phages. The English and grammar are clear and consistent throughout.

Specific points to address:

Lines 217-219 mentions that tail size is consistent with helper phage, yet lines 221 states that tail length is the same for Ando and AndoPrime. This is confusing, clarify that Ando does not carry tail structural proteins.

Figure 1 include what the scale bars represent on the image panels as well as in the legend to make it easier to understand.

**Associate Editor Recommendation: Minor Revision**

The reviewers were generally positive about the revised manuscript. Before acceptance, please address the two remaining points raised by Reviewer 1: clarify the description of Ando and AndoPrime so that it is clear that Ando does not encode tail structural proteins and that the observed tails are helper-derived, and label the scale-bar values directly within the Figure 1 panels as well as in the legend. These limited presentation and clarification issues should be readily addressed through minor revision.

**EIC Decision: Minor Revision**

**Author's Response to Decision Letter for (FEMSMC-2026-048.R1)**

Dear Dr. Scott,

Thank you and the reviewers for the thorough review process and constructive feedback. We have

addressed all remaining aspects.

We hope that the revised version is now suitable for further consideration in FEMS Microbes.

Thank you for your consideration.

Sincerely,

Tom Luthe and Julia Frunzke

**Reviews of manuscript FEMSMC-2026-048.R2**

**Associate Editor Recommendation: Accept**

Thank you for your careful attention to the reviewers’ comments. The manuscript has been strengthened and is now suitable for publication. Congratulations on the acceptance of your paper.

**EIC Decision: Accept**
