# Supplementary material for: A satellite in the phage universe: isolation and characterization of six novel Streptomyces phages AndoPrime, Ando, Arkania, Jaresh, Tatooine, and Galidraan
Source: FEMS Microbes. 2026 Sep 7;7:xtag048. doi: 10.1093/femsmc/xtag048 (PMC13587820; doi:10.1093/femsmc/xtag048)
Supplement: Supplementary file 1 [file xtag048_supplemental_files.zip › Kabuu_et al_Streptomyces phages_revised_SUPPLEMENT.docx]

Supplementary Information to

**A satellite in the phage universe: Isolation and characterization of six novel *Streptomyces* phages AndoPrime, Ando, Arkania, Jaresh, Tatooine, and Galidraan**

**Moses Kabuu^1,2^, Annika Ritzerfeld^3^, Biel Badia Roigé^1,2^, Samy Mechrouki^1,2^, Maximilian K Nocke^4,5^, Eva Davoudi^1,2^, Tom Luthe^1,4#^ and Julia Frunzke^1,2#^**

^1^Institute of Bio- and Geosciences, Biotechnology (IBG-1), Forschungszentrum Jülich, 52425 Jülich, Germany

^2^Faculty of Mathematics and Natural Sciences, Institute of Microbial Interactions, Heinrich-Heine-Universität Düsseldorf, 40225 Düsseldorf, Germany

^3^Department of Chemistry and Biotechnology, FH Aachen - University of Applied Sciences, 52428 Jülich, Germany

^4^Ruhr University Bochum, Medical Faculty, Department of Translational and Computational Infection Research (TRACiR), 44801 Bochum, Germany

^5^European Virus Bioinformatics Center (EVBC), Jena, Germany

^#^Corresponding authors: j.frunzke@fz-juelich.de; tom.luthe@ruhr-uni-bochum.de

**Supplementary Figure S1: Quality parameters of the multiple sequence alignment (MSA) of the 17 phage terminase proteins.** The total length of the MSA is 679 positions. All presented values are calculated by applying a sliding window with window size of 30 and step size of 10 to the MSA. **(A)** Coverage of sequences of the MSA. Blue indicates number of sequences with an amino acid at the respective position, while yellow indicates the number of sequences with gaps. **(B)** The Shannon entropy indirectly indicates diversity at the respective position of the MSA. Shades of red refer to the left Y-axis ignoring or including gaps. The lower the Shannon entropy, the lower the information content, hence the diversity. Conservation of the most frequent amino acid for each position is indicated in green and referring to the right Y-axis.
